# A 4-year follow-up of patients with medication-overuse headache previously included in a randomized multicentre study

**DOI:** 10.1007/s10194-010-0285-1

**Published:** 2011-01-05

**Authors:** Knut Hagen, Claus Albretsen, Steinar T. Vilming, Rolf Salvesen, Marit Grønning, Grethe Helde, Gøril Gravdahl, John-Anker Zwart, Lars Jacob Stovner

**Affiliations:** 1 Department of Neuroscience, Faculty of Medicine, Norwegian University of Science and Technology, Trondheim, Norway; 2 Department of Neurology, Faculty of Medicine, Norwegian National Headache Centre, St. Olavs Hospital, NTNU, 7491 Trondheim, Norway; 3 Department of Neurology, Tromsø University Hospital, Tromsø, Norway; 4 Department of Neurology, Oslo University Hospital, Ullevaal, University of Oslo, Oslo, Norway; 5 Department of Neurology, Nordland Hospital, Bodø, Norway; 6 Institute of Clinical Medicine, University of Tromsø, Tromsø, Norway; 7 Department of Neurology, Haukeland University Hospital, Bergen, Norway

**Keywords:** Medication-overuse headache, Follow-up, Outcome, Predictors

## Abstract

The aim of this study was to evaluate the long-term outcome in 61 patients with medication-overuse headache (MOH) who 4 years previously had been included in a randomized open-label prospective multicentre study. Sixty patients still alive after 4 years were invited to a follow-up investigation. Fifty patients (83%) participated. Sixteen visited a neurologist, 22 were interviewed through telephone, 2 gave response by a letter, and 10 were evaluated through hospital records. The influence of baseline characteristics on outcome 4 years later was evaluated by non-parametric tests. *p* values below 0.01 were considered significant. At follow-up, the 50 persons had a mean reduction of 6.5 headache days/month (*p* < 0.001) and 9.5 acute headache medication days/month (*p* < 0.001) compared to baseline. Headache index/month was reduced from 449 to 321 (*p* < 0.001). Sixteen persons (32%) were considered as responders due to a ≥50% reduction in headache frequency from baseline, whereas 17 (34%) persons met the criteria for MOH. None of the baseline characteristics consistently influenced all five outcome measures. Total Hospital Anxiety and Depression Scale (HADS) score at baseline was predictors (*p* < 0.005) for being a responder after 4 years. At 4 years’ follow-up, one-third of the 50 MOH patients had ≥50% reduction in headache frequency from baseline. A low total HADS score at baseline was associated with the most favorable outcome.

## Introduction

The prevalence of medication-overuse headache (MOH) is 1–2% in the general population [1–6]. Although MOH is a common problem worldwide [7], no established consensus for treatment strategies exists [8]. Scientific studies on MOH have accumulated during the past years, but conclusions as to the best treatment is scarce due to differences in definition of MOH, therapeutic approaches, types of primary headache, and study design [8]. After treatment, relapse rates of chronic headache (≥15 days/month) combined with medication overuse are typically high. Relatively few follow-up studies have evaluated the long-term outcome of MOH [9]. Very few studies have published follow-up data beyond 12 months, most of them were performed before the introduction of the International Classification of Headache Disorders, 2nd edn (ICHD-II) [10]. Summarizing data of six follow-up studies, a mean relapse rate of 26% during the first year was found [9], whereas follow-up studies of longer duration have reported relapse rates between 20 and 60% [11–17]. The knowledge about predictors is limited, but in some previous long-term follow-up studies the prognosis has been reported to be better, e.g., in women, migraine patients, and in those with short duration of medication overuse [13–16].

Previously, we performed a randomized open-label 1-year follow-up study of MOH patients diagnosed according to the revised ICHD-II criteria of MOH [18, 19]. A main finding was that early introduction of preventive treatment without a previous detoxification program reduced the suffering of total headache more abruptly than with just withdrawal [18]. In the last part of the 1-year follow-up, all included patients were offered the treatment considered to be optimal for them, preventive treatment included. However, whether a high focus on prophylactic treatment during the first year could prevent a suggested high relapse rate was unclear, mainly because very few prospective long-term studies of MOH patients using the revised ICHD-II criteria have been performed.

Therefore, the main purpose of the present study was to evaluate the 4-year follow-up outcome in the group of MOH-patients that previously were included in the randomized open-label 1-year follow-up. A secondary goal was to evaluate the influence of baseline characteristics on outcome 4 years later.

## Methods

The study was approved by the Regional Committee for Ethics in Medical Research, the Norwegian Data Inspectorate, and was registered at ClinicalTrials.gov (number NCT00918671).

### Patients

Sixty patients aged 22–64 years were in the period between January 2008–May 2010 invited to a 4-years’ follow up. All had 4 years previously participated in a prospective, open-labeled multicenter study [18], and at inclusion time they all fulfilled the revised ICHD-II criteria of MOH [19].

**Fig. 1:**
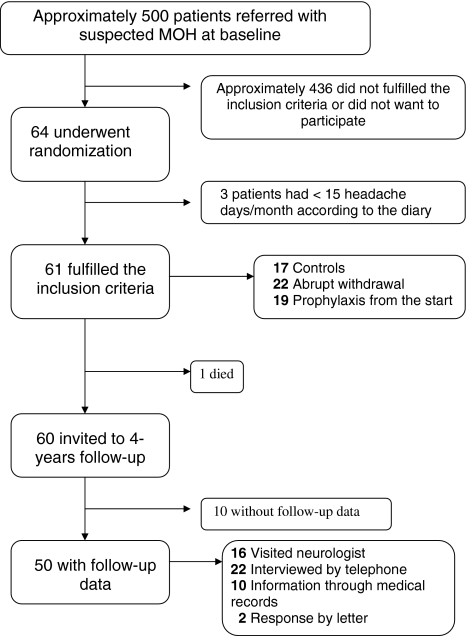
Study flow diagram

The initial study is described in detail previously [18]. Briefly, the majority of patients were included at St. Olav’s Hospital, where on average 13% of those referred with suspected MOH were included in the study [18]. Presumably, approximately 500 patients with suspected MOH were screened during the inclusion period between 2004 and 2006 (Fig. 1). At baseline the patients were randomly assigned to three different groups; (a) abrupt withdrawal with start of preventive treatment after 3 months, (b) preventive treatment from day 1 without abrupt withdrawal, (c) controls without preventive medication or abrupt withdrawal. Follow-up visits were scheduled at months 1, 3, 5, and 12 after inclusion. The controls finished the study period after 5 months’ observation, but were then offered the treatment considered to be optimal for them (withdrawal or prophylactic treatment) and further follow-up visits. After the end of the 1-year follow-up, no further regular visits were offered by the neurologists. The primary care physician (PCP) might switch medication and treatment according to their best clinical judgment at any time, or refer the patient to a neurologist for new consultations.

### Study procedure

Individuals living near Trondheim who agreed to participate were offered a consultation by a neurologist (KH) including a semi-structured interview. Prior to the visit they had to complete a headache diary for at least 1 month. In the headache diary patients recorded daily whether they had headache or not, and if they had headache, its duration when they were awake, nausea, brand name and number of doses of acute headache medication, and absence from work. The headache severity was scored on a 3-point scale from 1 to 3, explained in the diary as: 1 = mild; does not inhibit work or other activities, 2 = moderate; inhibits but does not exclude work or other activities, 3 = severe; excludes work or other activities.

Individuals living distant to Trondheim agreeing to participate, were interviewed through telephone by neurologist (KH) using the same semi-structured interview, concerning headache frequency, headache intensity, headache duration, use of acute medication, use of prophylactic treatment, and absence from work. Patients interviewed through telephone or face-to-face were asked to answer questions in the Hospital Anxiety and Depression Scale (HADS) and a health-related quality of life-questionnaire (HRQqL) using the Short Form (SF)-12 [20, 21]. The SF-12 measures HRQoL in two main domains, a mental health component score (MCS-12) and a physical health component score (PCS-12).

Medical records were checked for individuals who did not respond to the invitation letter. If they had visited a neurologist for headache after the 1-year follow-up had finished, information about headache frequency and use of medication during the last consultation was collected.

### Outcome measures

The primary outcome measure was the change in number of headache days/month from the baseline period to the 4-year follow-up. The secondary outcome measures were change in days with use of acute headache medication/month, mean headache hours, headache index (HI)/month (sum of the products of “headache days/month” combined with “mean daily hours with headache” and “mean daily headache severity” on days with headache), sick leave days/month, anxiety and depression measured by HADS, and SF-12 in two main domains MCS-12 and PCS-12. At 4-year follow-up, we also estimated number of: (a) responders defined as those with ≥50% reduction in headache days/month compared to baseline and being without medication overuse, and (b) patients with headache ≥15 days per month combined with medication overuse.

### Outcome predictors

The influence of the following baseline characteristics on outcome was evaluated: sex, age, education level, employment status, number of years with headache, type of intervention in the original study (3 groups), original headache diagnosis, having tried at least two types of preventive medication prior to randomization, MCS-12, PCS-12, and HADS (total, depression, and anxiety) score.

We also evaluated whether surgery or onset of medical conditions like, e.g., depression, or diabetes mellitus type 2 during the follow-up influenced the outcome.

### Statistics

Non-parametric tests were used for comparisons between groups (Kruskal–Wallis test, Mann–Whitney and Chi-square test) and within groups (Wilcoxon test) because of skewed distribution of data. Correlations were evaluated by Spearman’s rho. More than ten different predictive factors were evaluated. Multiple comparisons can be associated by type I error, but the highly conservative Bonferroni-type corrections can be associated with type-II errors [22]. As a compromise, *p* values below 0.01 were considered significant.

When appropriate, the influence of predictors was also evaluated in multivariate analyses, using logistic regression (dependent variables: responders or MOH) or linear regression (dependent variables: headache days/month, HI/month, or medication days/month). Adjustments were made for the most important predictors identified by the non-parametric tests (i.e., gender, headache diagnosis, HADS-total, PCS-12, and years with headache). Analyses were carried out using SPSS version 16.0 for windows (SPSS Inc., Chicago, IL, USA).

## Results

In the original study population of 64 randomized patients, 42 (66%) were included at St. Olav’s Hospital, whereas a total of 22 (34%) were included from the other hospitals.

Sixty-one patients met the inclusion criteria of MOH (Fig. 1). The remaining three used triptans >10 days per month, but had less than 15 headache days/month at baseline. During the 1-year follow-up one committed suicide, but according to the patient’s GP this was not related to headache.

Among the 60 participants who met the inclusion criteria of MOH and were still alive 4 years after randomization, follow-up data of headache status were available in 50 subjects (83%), whereof 16 visited a neurologist, 22 were interviewed through telephone, and two gave response by letter (Fig. 1). In ten patients, headache data were available in their hospital records. Information about headache was collected on average 4.0 years (95% CI 3.7–4.2 years) after randomization. The duration from baseline to end of follow-up was slightly longer (*p* = 0.13) for individuals who visited a neurologist (mean of 4.2 years) compared to those interviewed through telephone (4.0 years) and to those with headache data available in medical records (3.5 years) (Table 1).

**Table 1:** Characteristics of the 60 participants related to examination methods at follow-up

	Visited neurologist	Telephone or letter	Medical records	No information available	Statistics
Number (*n* = 60)	16	24	10	10	
Women (%)	44	71	60	70	0.34*
Change in headache days/month at month 5 versus baseline	−1.4	−4.6	−3.3	−9.3	0.22^†^
Change in headache index/month at month 5 versus baseline	3.4	−44.4	−42.0	−92.9	0.79^†^
Tried less than two preventive drugs at baseline (%)	63	67	70	90	0.48*
Mean number of years from baseline to end of follow-up (95% CI)	4.2 (3.9–4.5)	4.0 (3.9–4.1)	3.5 (2.5–4.5)	0.8 (0.4–1.1)	*p* < 0.001^†^

* Chi-square test

^†^Between group analyses: Kruskal–Wallis test

### Participants versus non-participants

Among the ten persons (17%) without available long-term follow-up data, the last consultation by a neurologist was performed 8.3 months (mean) after randomization (SD 3.9). Compared to the 50 patients who participated in the follow-up, these ten persons experienced somewhat more prominent reduction in headache days/month (−3.3 vs. −9.3 days/month, *p* = 0.07) and headache index/month (−29 vs. −93, *p* = 0.36) estimated 5 months after randomization (Table 1). However, none of these differences were statistically significant.

### Outcome

**Fig. 2:**
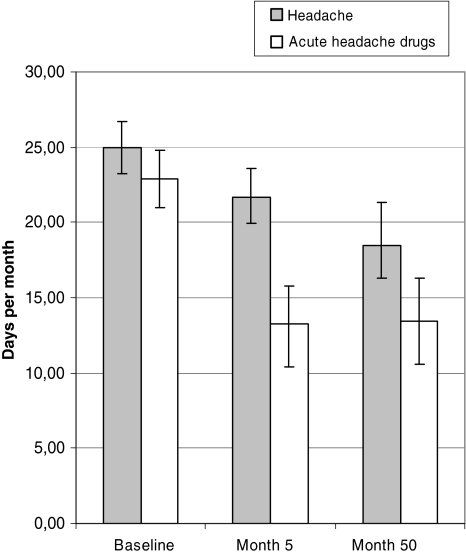
Headache days/month and days with acute headache drugs/month with 95% confidence intervals at baseline, 5, and 50 months

At follow-up the group of 50 individuals had a significant reduction in headache days/month, days with use of acute headache medication/month (Fig. 2), headache index/month, and days with sick leave compared to baseline, more prominent at 4-year follow-up than at month 5 (Table 2). Also, physical health component score (PCS-12) and mental component score (MCS)-12 increased significantly during the 4-years follow-up for the 38 individuals with complete data of SF-12 (Table 2).

**Table 2:** Efficacy variables (with 95% confidence intervals) at baseline, 5 months and 4 years’ follow-up

	Baseline	5 months	4 years
Headache days/month (*n* = 50)	25.0 (23.2–26.7)	21.6 (18.9–24.4)*	18.4 (15.1–21.8)***
Days with acute headache drugs/month (*n* = 50)	22.9 (21.0–24.8)	13.3 (10.4–16.3)***	13.4 (10.3–16.4)***
Headache hours/month (*n* = 50)	8.1 (6.8–9.4)	8.3 (7.1–9.6)	7.7 (6.2–9.2)
Headache index/month (*n* = 50)	449 (349–548)	420 (306–534)	320 (224–418)***
Days with sick leave/month (*n* = 35)	6.3 (1.7–11.0)	2.2 (0.3–4.2)*	0.9 (0.2–1.6)**
Mental health component (MCS-12) (*n* = 38)	51.2 (44.6–57.7)	57.9 (50.8–64.9)**	62.7 (53.8–71.6)**
Physical health component (PCS-12) (*n* = 38)	46.7 (40–53.1)	51.0 (43.5–58.5)*	59.8 (51.0–68.6)**
Number of prophylactic drugs tried, mean (*n* = 50)	1.2 (0.8–1.6)	1.9 (1.4–2.3)***	2.5 (2.0–3.0)***

*** *p* ≤ 0.001; ** *p* ≤ 0.01; * *p* < 0.05

After 4 years, 20 persons (40%) had headache <15 days/month, whereof 16 (32% of the total group) had a ≥50% reduction in headache frequency compared to baseline. Thirty individuals (60%) had headache ≥15 days/month. Among these, 17 (34% of the total group) still had MOH, whereas the remaining13 persons did not overuse acute headache medication. The overused drugs were triptans (*n* = 7), simple analgesics (*n* = 5), or combination analgesics (*n* = 5).

At follow-up, 20 individuals (40%) were still using headache preventive medication (7 persons with MOH and 13 without MOH). The most common drugs were angiotension II blockers (*n* = 6) and/or amitriptyline (*n* = 6), followed by β-blockers (*n* = 4), topiramate (*n* = 3), and gabapentin (*n* = 1). During the time to follow-up, some type of medical event was recorded in 15 (30%) out of the 50 participants; onset of depression (*n* = 2), diabetes mellitus type 2 (*n* = 2), myocardial infarction (*n* = 1), some type of surgery (*n* = 8), alcohol abuse (*n* = 1), and Graves’disease (*n* = 1).

### Predictors of outcome

The influence of baseline characteristics on the long-term outcome evaluated by non-parametric tests is shown in Table 3. None of them influenced all five outcome measures, and no significant predictors were found for headache days/month at 4-years’ follow-up. The strongest inverse correlation was found between baseline values of total HADS score and HADS-anxiety score and being a responder. A significant inverse correlation was also found between PCS-12 and medication days/month after 4 years (Table 3). No significant correlation was found between type of intervention during the first months of follow-up and outcome at 4-years follow-up.

**Table 3:** The influence of baseline predictors of outcome at 4-years follow-up expressed by *p* values evaluated by non-parametric tests

Predictors	Headache days/month	Headache index/month	Medication days/month	Responders	MOH
	*p* value	*p* value	*p* value	*p* value	*p* value
Male	0.019^b^	0.035^b^	0.051^b^	0.14^a^	0.18^a^
Age	0.85^c^ (0.03)	0.65^c^ (0.07)	0.86^c^ (0.03)	0.22^b^	0.56^b^
Years with headache	0.03^c^ (0.31)	0.18^c^ (0.19)	0.04^c^ (0.29)	0.23^b^	0.045^b^
Type of intervention (3 groups)	0.41^b^	0.84^b^	0.73^b^	0.85^a^	0.36^a^
>2 preventive drugs	0.63^b^	0.71^b^	0.19^b^	0.52^a^	0.42^a^
MCS-12	0.41^c^ (0.12)	0.26^c^ (0.16)	0.67^c^ (0.06)	0.31^b^	0.46^b^
PCS-12	0.04^c^ (0.29)	0.027^c^ (0.31)	0.007^c^ (0.38)	0.10^b^	0.19^b^
HADS-anxiety	0.04^c^ (0.29)	0.0125^c^ (0.32)	0.14^c^ (0.21)	0.004^b^	0.15^b^
HADS-depression	0.06^c^ (0.26)	0.0136^c^ (0.30)	0.057^c^ (0.27)	0.012^b^	0.22^b^
HADS-total	0.027^c^ (0.31)	0.013^c^ (0.35)	0.056^c^ (0.27)	0.003^b^	0.17^b^
Type of drug overused	0.52^b^	0.28^b^	0.51^b^	0.91^a^	0.77^a^
Headache diagnosis^d^	0.02^b^	0.006^b^	0.12^b^	0.027^a^	0.82^a^
Employed, full-time or part-time	0.08^b^	0.06^b^	0.25^b^	0.14^a^	0.18^a^
≤9 years of education	0.82^b^	0.32^b^	0.06^b^	0.54^a^	0.04^a^
Surgery during follow-up	0.42^b^	0.18^b^	0.26^b^	0.83^a^	0.59^a^

^a^Chi square test

^b^Kruskal–Wallis test

^c^Spearman’s rho with correlation coefficient in brackets

^d^The original headache before they developed MOH at baseline: 18 subjects had pure migraine, 17 tension-type headache without migrainous features, and 15 mixed headache

In a multivariate analysis the correlation between HADS-anxiety score/total HADS score and being a responder (adjusted for headache diagnosis), and between PCS-12 and medication days/month remained statistically significant (adjusted for gender, education level, and years with headache) (*p* < 0.007).

## Discussion

In this 4-year follow-up study, one-third of the MOH patients still had ≥50% reduction in headache frequency from baseline, and two-thirds were without medication overuse. None of the baseline characteristics consistently influenced all outcome measures.

There are, however, some limitations to consider with the present study. As for all clinical-based studies, one may question to what degree the main results can be generalized. In the initial 1-year follow-up study, less than 15% of patients referred with suspected MOH were included [18]. The majority of these did not fulfill the MOH diagnosis, and only a few of those who fulfilled MOH diagnosis declined to participate. It may also be relevant to point out that the non-participants in the 4-year follow-up had somewhat greater reduction in headache days/month 5 months after randomization than those who participated. Hence, our study population may represent individuals with a particular high headache burden, which may at least partly explain why the burden of headache was still relatively high after 4 years. Thus, selective participation could not be ruled out, and generalization of the results should be done with some caution, because selective participation could not be ruled out.

Moreover, many patients did not fill in a headache diary on a regular basis, and those who visited a neurologist completed a headache diary only for 1 month. Although all participants also were asked about use of acute headache medication and headache frequency during the last 3 months, we cannot rule out that recall bias may have influenced our results. Ideally, to verify the diagnosis of MOH, all participants should have visited a neurologist at 4 years follow-up completing a 3-month headache diary prior to the visit. However, we had to use other methods of assessments for those who recently had consulted a neurologist, moved abroad, or lived far from Trondheim. Using this pragmatic strategy, a high participation rate (83%) was achieved, but makes the interpretation of the results more complicated.

Because study design, diagnosis of MOH, and treatment strategies differ widely between follow-up studies, direct comparisons of results should be done with caution. In an Austrian study, one-third of patients were considered to have a favorable outcome defined as <8 headache days/month and no drug overuse 5 years after withdrawal [16], which is similar to the responder rate of 32% at the end of follow-up in the present study. In contrast, a much higher responder rate was reported by Andrasik et al. (78%) and Diener et al. (66%) [11, 23]. However, as already mentioned, the present study may to some degree be biased toward poor outcome due to participation bias, because the non-participants in the 4-year follow-up had somewhat more prominent reduction in headache days/month 5 months after randomization. Thus, we cannot rule out the possibility that the responder rate could have been higher if all 60 invited persons had participated. Anyway, our relapse rate of 34% is in fact somewhat lower than the mean relapse rate of 41% (range 21–60%) reported in seven previous studies with a follow-up of at least 4 years (Table 4) [11–17]. The prognosis was even better for 109 MOH patients identified in a population-based survey. In a 1½-year follow-up of these patients, 24% still had MOH, whereas 42% did not experienced chronic headache [24].

**Table 4:** Outcome in seven studies with follow-up ≥ 4 years

Publication year (reference)	Dropout rate (%)	Headache days/month baseline	Duration of follow-up (years, mean)	Headache days/month Follow-up	Responder rate^1^ (%)	Relapse rate (%)	Statistical significant predictors
1996 [13]^Ba^	30	–	5	–	50^A^	40	Number of tablets, duration of drug abuse
1999 [15]^Ba^	61	–	5.9	12.3	–	21	Sex, type of overused drugs
2001 [16]^Bd^	12	–	4	–	–	60	Number of tablets, duration of drug abuse
2001 [12]^Bd^	–	–	9.3	–	35^1^	33	None
2004 [14]^Ba^	22	–	4	–	–	45	Headache type, type of overused drugs
2009 [11]^Bb^	45	26.1	5	11.3	78	–	None
2010 [17]^Bc^	6	–	4	–	–	44	Remission at year 1
Mean	29	26.1	5.3	11.8	54	41	

^A^Headache ≤8 days or less without medication overuse

^B^MOH diagnosis based on: (a) First version of the International Headache Society (IHS) from 1988 (ICDH-1), (b) criteria proposed by Silberstein and Lipton in 2000, (c) ICDH-2 or later versions, or (d) other criteria or not clearly stated

In the present study, most of the patients did not have regular contact with their own PCP or neurologist which at least to some degree, explain why the burden of headache was still high after 4 years with a mean of 18.4 headache days/month and HI/month of 321. However, compared to the situation 5 months after inclusion in the study, the number of headache days/month, days with sick leave, and headache index/month were reduced after 4 years. Accordingly, the PCS-12 and MCS-12 increased significantly during the 4-years’ follow-up for those with complete SF-12 data. A similar pattern with sustained improvement was reported by Andrasik et al. in a 5-year follow-up [15]. Possibly, a high focus on the use of preventive treatment during the first year of follow-up may have resulted in a sustained improvement. However, we cannot rule out that spontaneous improvement, or “regression-to-the-mean”, at least in part, may explain the favorable course.

In the present study, 60% with MOH at baseline still had chronic headache (≥15 days/month) at 4-year follow-up. The corresponding long-term prognosis of primary chronic headaches without medication overuse is largely unknown. Interestingly, however, among individuals with chronic TTH or chronic migraine at baseline in 1989, 56% still had chronic headache in 2001 in a Danish population-based follow-up study [25].

None of the evaluated baseline predictors had an impact on all five outcome measures. It should be emphasized that we used more conservative statistical methods than most of the previous long-term follow-up studies, and the chosen significance level (*p* values below 0.01) could be associated with a type II error. Although women and individuals with “pure” migraine tended to have more prominent decrease in headache days/month at 4-years follow-up, a *p* value below 0.01 was not achieved. Thus, we could not statistically confirm a better prognosis in women and migraine patients which was reported in previous long-term follow-up studies [14, 15].

In our study, the most favorable outcome was found for individuals with low total HADS score. In addition, a significant inverse correlation was also found between PCS-12 and medication days/month after 4 years. Few other follow-up studies have evaluated the influence of HADS score and quality of life at baseline on long-term outcome. In a 1-year follow-up of MOH patients, self-reported bodily pain measured by SF-36 was associated with poor outcome, whereas HADS score did not influence significantly [26].

Regarding relapse, no significant predictors were found. Number of years with headache did not significantly correlate with the diagnosis of MOH at follow-up. Of relevance, relapses have been associated with duration of medication overuse in two other long-term follow-up studies using a *p* < 0.05 as a significance level [13, 16]. Unfortunately, information about duration of medication overuse at inclusion was not available in the present study. Similarly, the total number of tablets overused was not measured at baseline, which previously has been reported to be associated with relapse at follow-up [13, 16]. We did not find that relapse was associated with type of drugs overused as reported by others [14, 15]. It should be emphasized that the statistical power was relatively low [18], and our analyses were based on a lower number of participants (*n* = 50) than the other comparable long-term follow-up studies lasting at least 4 years (mean number of participants = 70, range 38–101) [11–16].

With regard to type of intervention no significant difference in headache days/month was found. However, because all patients had access to the treatment considered to be optimal for them after the first year, no long-term differences between the groups should be expected.

A total of 17 individuals met the diagnosis of MOH at 4 years’ follow-up. However, it may be questionable whether all these have MOH in a pathophysiological sense. This consideration is of relevance for those who performed successful withdrawal, but did not experience headache improvement. Two patients in the withdrawal group (with the original diagnosis of tension-type headache) did not use pain killers the first 5 months of follow-up, but still had headache almost every day. However, at 4 years’ follow-up they fulfilled the MOH diagnosis due to overuse of acute medication, because headache improvement after discontinuation of medication overuse is no longer mandatory [19].

In conclusion, the initial type of intervention did not influence the outcome at 4-year follow-up. The long-term prognosis is relatively favorable as there was a continuing decline in headache, one-third of the MOH patients having ≥50% reduction in headache frequency from baseline, and two-thirds being without medication overuse. However, the fact that the total burden of headache was still high after 4 years in this group calls for large and scientifically well-designed intervention studies with long-term follow-up to obtain better treatment regimes for these patients.
